# A novel cone beam-CT approach for quantifying maxillary changes following secondary alveolar bone grafting in unilateral cleft patients

**DOI:** 10.1186/s12903-025-05666-3

**Published:** 2025-02-23

**Authors:** Basel Khalil, Tobias Regnstrand, Reinhilde Jacobs

**Affiliations:** 1 Section of Oral Radiology, Department of Dental Medicine, Division of Oral Diagnostics and Rehabilitation, Karolinska Institute, Huddinge, Sweden; 2 OMFS IMPATH Research Group, Department of Imaging & Pathology, Faculty of Medicine, KU Leuven & Oral and Maxillofacial Surgery, University Hospitals Leuven, Leuven, Belgium

**Keywords:** Cone beam computed tomography (CBCT), Maxillary changes, Secondary alveolar bone grafting (SABG), Cleft lip and/Or palate (CL/P), Quantitative assessment, Orthogonal planes

## Abstract

**Objectives:**

To quantify longitudinal bone changes in the maxilla after secondary alveolar bone grafting (SABG) in patients with unilateral cleft lip and/or cleft palate (CL/P), and to describe the maxillary dimensions before and after SABG in all orthogonal planes using a Cone Beam Computed Tomography (CBCT)-based methodology.

**Methods:**

Ethical approval was obtained to retrospectively analyze CBCT scans of children treated for unilateral alveolar clefts. Inclusion criteria encompassed individuals who underwent SABG before the eruption of the cleft-side permanent lateral incisor or, if absent, the permanent ca-nine, and had both preoperative and postoperative CBCT scans. A total of seven measurements of maxillary dimensions in all orthogonal planes, as well as in panoramic reconstruction, were conducted in the CBCT images. The normality of the data was confirmed with the Shapiro-Wilk test. A paired t-test was applied to assess significant differences between the preoperative and postoperative measurements (*p* < 0.05). The impact of age, gender, and their interrelation was evaluated by two-way ANOVA (*p* < 0.05).

**Results:**

A total of 47 patients, comprising 32 males and 15 females, were selected based on the specified criteria. Notably, we observed a significant increase in sagittal, vertical, and transverse dimensions between the preoperative scans and those taken one year postoperatively (*p* < 0.05), irrespective of the patients’ ages and genders. When considering gender, data indicated that male patients exhibited wider transverse dimensions in both the preoperative and postoperative measurements.

**Conclusions:**

This study introduced a CBCT-based method to quantitatively assess maxillary changes in unilateral CL/P patients after SABG. The approach demonstrated continuous dimensional changes in all orthogonal planes. In addition, it described the maxillae dimensions in all planes. Future research can utilize this method for precise measurement of maxillary alterations and dimensions.

## Introduction

**Fig. 1:**
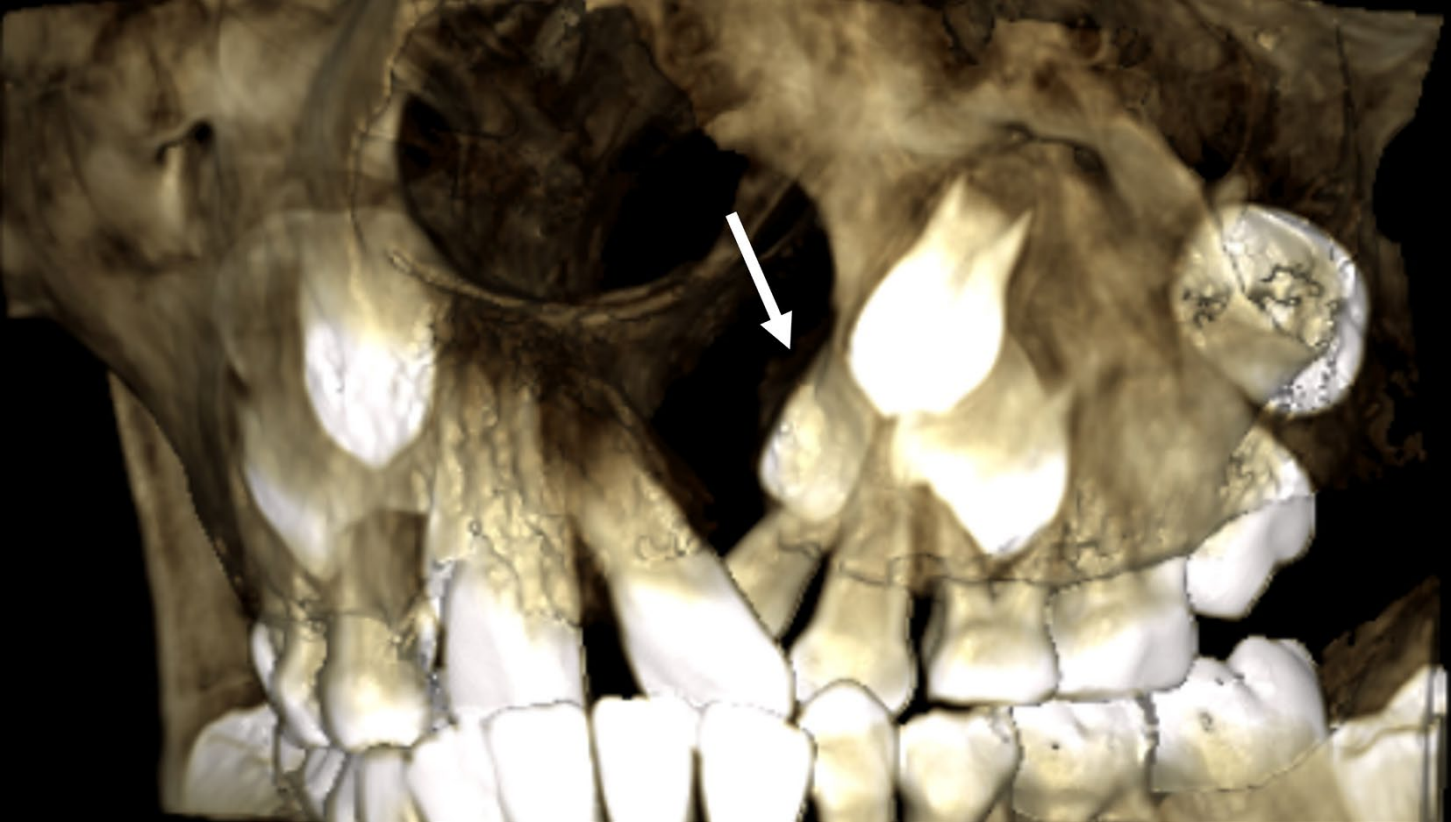
A 3D volume rendering, derived from a preoperative CBCT scan, depicts an alveolar cleft before the eruption of the permanent lateral incisor (white arrow) and canine on the cleft side

Cleft Lip and/or Cleft Lip Palate (CL/P) are the most common congenital facial deformities, with a prevalence of 0,13% [1]. An alveolar cleft is a deformity within the maxillary alveolar bone that should normally embed upper lateral incisor and canine (Fig. 1). Alveolar clefts are present in almost three out of four CL/P cases [2]. This deformity may manifest unilaterally or bilaterally. Permanent lateral incisors and canines usually erupt between 7 and 11 years old. This implies that an alveolar cleft may hamper dentoalveolar growth, tooth eruption and related periodontal tissue development in the upper maxilla [3]. Therefore, an alveolar cleft is surgically repaired through bone grafting to stabilize the maxillary arch, facilitate the eruption of the lateral incisor and canine, support the teeth adjacent to the cleft, elevate the alar base of the nose, and prevent oronasal fistulas [4, 5]. Primary bone grafting, performed during primary dentition has been observed to impede the growth of the midfacial complex [6]. Correspondingly, most cleft centers follow the standard protocol introduced by Boyne and Sands in 1972, where alveolar bone grafting is performed in mixed dentition prior to the eruption of the permanent canine. This procedure is commonly referred to as secondary alveolar bone grafting (SABG) [7]. However, delaying SABG prior to the eruption of the permanent canine may jeopardize the eruption of the adjacent permanent lateral incisor [8]. Therefore, SABG should also aim to enable the eruption of the cleft-side lateral incisor when presented [9, 10].

To assess alveolar clefts before and after bone grafting in the light of multidisciplinary rehabilitation, diagnostic imaging plays a key role. Intraoral radiographs have been traditionally used for radiological examinations [11, 12]. Similarly, cephalometric imaging has been routinely used as a modality for describing growth and facial development in CL/P [13]. Over the past decade, the use of cone-beam computed tomography (CBCT) in CL/P care has significantly increased [14–17]. This state-of-the-art technique allows for a more precise examination of the alveolar cleft, and enables a more accurate evaluation of the bone graft’s outcome, albeit at the expense of higher radiation exposure [18, 19]. However, the use of CBCT examinations for the cleft area is widely considered justified [15, 20]. Therefore, the use of CBCT for both preoperative examination and postoperative outcome assessment in cases of cleft conditions is strongly recommended [14, 15, 20]. Furthermore, following the optimized imaging protocol for CL/P patients proposed by De Mulder et al. [15], a low-resolution CBCT scan of both jaws with a field of view (FOV) typically measuring 12 × 15 cm² is routinely recommended before conventional orthodontic treatment, which follows the SABG, and again upon completion of treatment.

While previous studies have primarily focused on three-dimensional (3D) assessment of bone defects and bone augmentation in cleft care using CBCT imaging [16, 21], there is a noticeable gap in research regarding the application of 3D evaluation for examining the bone changes in CL/P. The objective of this study is to quantify maxillary changes after SABG by employing a methodology involving specific dentoalveolar and skeletal CBCT-based measurements. Our secondary aim is to define the dimensions of the maxilla in all orthogonal planes for this specific patient category, both before and after SABG. The null hypothesis asserts that there is no significant difference in maxillary dimensions before and after SABG in patients with unilateral CL/P, when measured by this 3D approach.

## Materials and methods

### Data collection

The sample size for this study was determined using a prior power analysis. This analysis was conducted with G*Power software (version 3.1.9.7), based on findings from a similar study [22]. The power analysis indicated that a minimum of 41 participants would be required to achieve statistically significant results, with a power of 80% and an alpha level of 0.05. This calculation ensures that the study is adequately powered to detect meaningful differences or effects, thereby minimizing the risk of Type II errors.

This retrospective follow-up study was conducted at the Stockholm Craniofacial Centre, Karolinska University Hospital in Sweden, including patients with non-syndromic unilateral CL/P affecting the alveolar process. The standard surgical protocol for clinical cleft treatment involves autogenous bone grafting from the iliac crest, ideally performed before the eruption of the cleft-side lateral permanent incisor or, if absent, the permanent canine. Patients aged between 7 and 11 years who underwent SABG and had both preoperative and postoperative CBCT scans available were included.

The rationale for selecting patients within this age range was based on the typical timing of lateral incisor and canine eruption in growing individuals. To maintain a homogeneous dataset and ensure a cohort of patients at a similar growth stage, those who underwent surgery after canine eruption were excluded. This exclusion criterion minimizes potential confounding factors related to different growth stages, thereby enhancing the validity and reliability of the study’s findings.

**Fig. 2:**
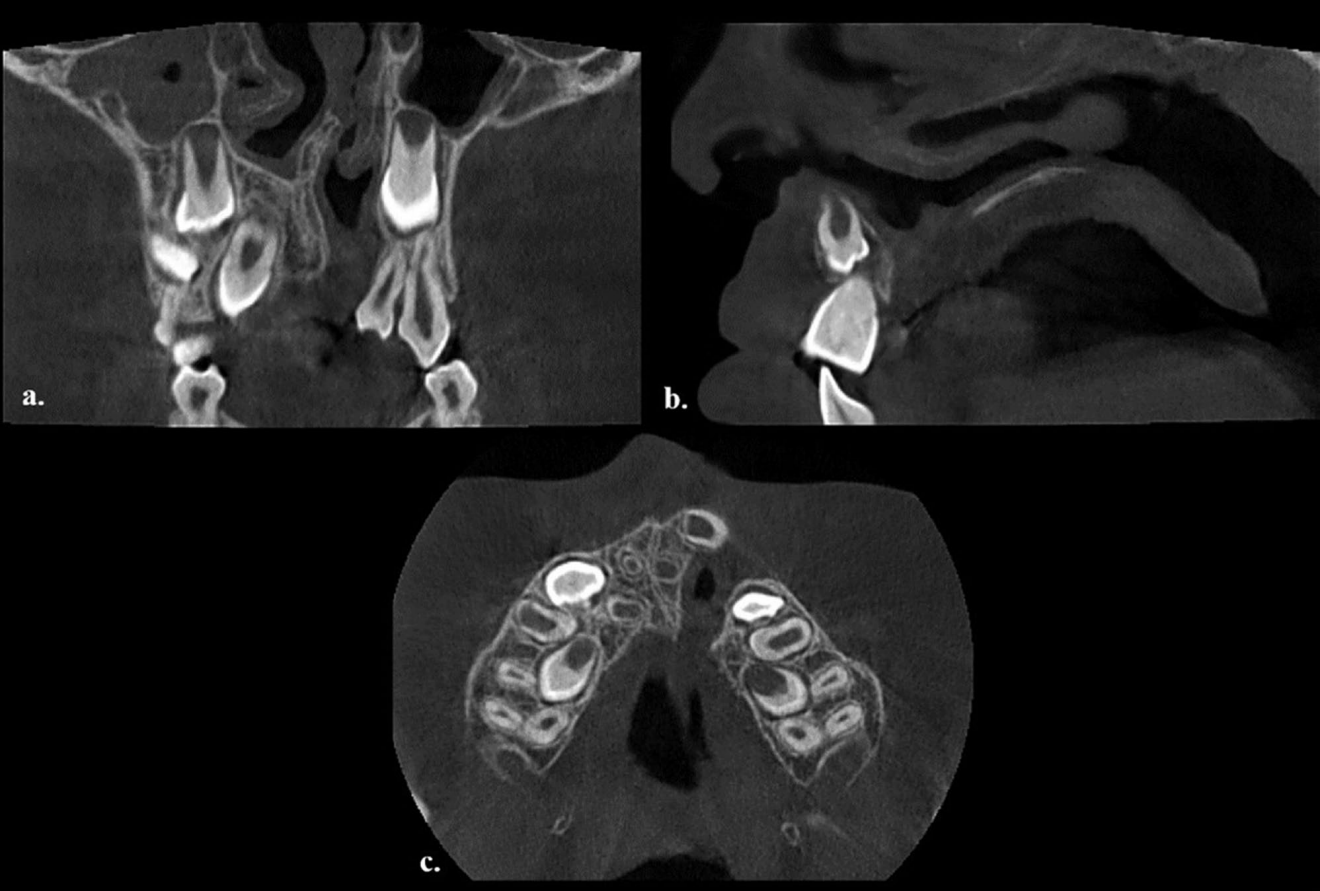
Preoperative CBCT scan obtained with an 8 × 5 cm² field of view (FOV) configuration, (**a**) transverse, (**b**) sagittal, (**c**) axial

The initial CBCT scans were acquired before surgical intervention to evaluate the anterior maxillary anatomy and the eruption of the cleft-side permanent lateral incisor and canine Fig. 2. Postoperative CBCT scans were taken approximately one year after the preoperative scans to assess the outcome of bone grafting. The retrospective nature of this study ensured that no additional radiation exposure occurred.

Patients with transverse collapse and severe teeth misalignment received pre-graft orthodontic intervention, including transverse maxillary expansion or removable appliances, to address the collapse and optimize dental arch and tooth conditions. Exclusion criteria for the study: CBCT scans of insufficient quality, lack of CBCT follow-up data, and CBCT data of SABG performed after canine eruption.

### Image acquisition

CBCT scans were acquired with a Promax 3D Mid scanner (Planmeca OY, Helsinki, Finland). The selected imaging parameters included a tube voltage of 90 kV, tube current ranging from 4.5 to 8 mA, exposure time of either 4–12 s, voxel size of 0.2 mm, and with an 8 × 5 cm^2^ FOV. The acquired data were subsequently saved and preserved as Digital Imaging and Communications in Medicine (DICOM) files.

### Anatomical landmarks

**Fig. 3:**
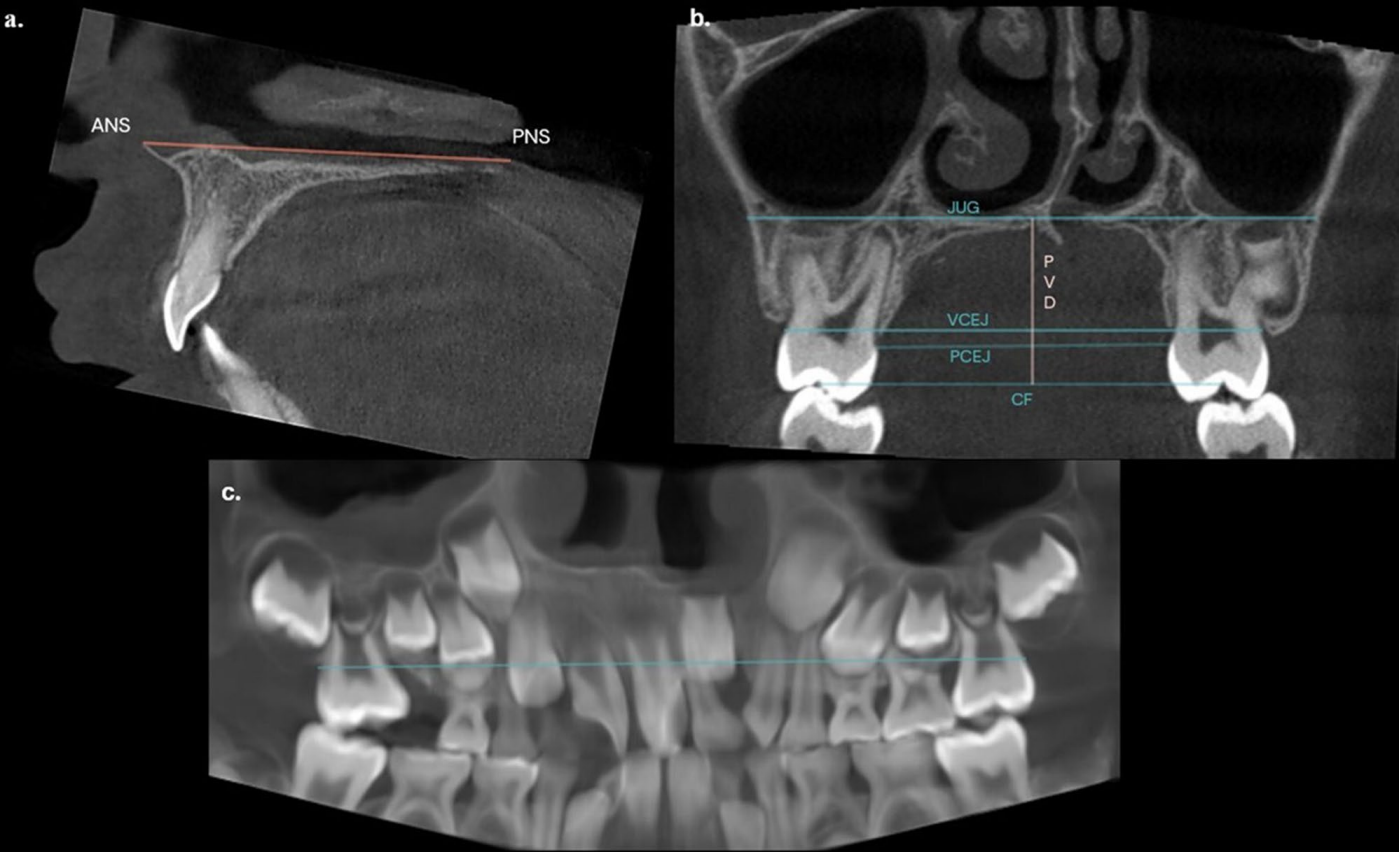
Maxillary dimensional measurements on CBCT slices and panoramic reconstruction **a**) Sagittal plane, ANS-PNS: anterior nasal spine- posterior nasal spine distance. **b.)** Transverse plane PVD: palatal vault depth, CF: maxillary first molar’s central fossa distance, PCEJ: palatal cementoenamel junction (CEJ) distance, VCEJ: vestibular CEJ distance, JUG: jugular distance. **c.)** Panoramic view, AL: arch length distance. The panoramic view was meticulously reconstructed, by manually tracing the CEJ of the presented permanent teeth

A total of seven linear measurements were conducted between predetermined anatomical landmarks. The assessment involved measuring the distance between the following anatomical structures: anterior nasal spine-posterior nasal spine (ANS-PNS), palatal vault depth (PVD), bilateral maxillary first molar’s central fossa (CF), bilateral palatal cementoenamel junction (CEJ) (PCEJ), bilateral vestibular CEJ (VCEJ), bilateral jugular (JUG) and bilateral arch length (AL) (Fig. 3). Measurements in the transverse plane (CF, PCEJ, VCEJ, JUG) and panoramic view (AL) were divided into cleft and normal sides. This division utilized a vertical line passing through the midpalatal suture as a reference line to compare the cleft side to the normal side at the level of maxillary first permanent molars [23]. The midpalatal suture was the best option since the CBCT scans were conducted with a small FOV, making it impossible to use cranial structures as a reference. For clarity, transverse measurements were either dentoalveolar-based (CF, PCEJ, VCEJ) or skeletal-based (JUG). For a detailed description of landmarks, dentoalveolar and skeletal distances, refer to Table 1.

**Table 1:** Description of landmarks and distances for assessing maxillary dimensions

	Landmark measurements	Plane	Description
1	ANS-PNS distance	Sagittal	Distance between ANS and PNS
2	Cleft side: central fossa to midline CCF Normal side: central fossa to midline NCF Total: maxillary first molar central fossa CF	Coronal Coronal Coronal	Distance between the central fossa of the maxillary first permanent molar in the cleft-side and the midline Distance between the central fossa of the maxillary first permanent molar in the normal-side and the midline Distance between the central fossa of the maxillary first permanent molars
3	Palatal vault depth PVD	Coronal	Distance between the inferior border of the midpalatal suture and the line between the central fossa CF
4	Cleft side: palatal CEJ to midline CPCEJ Normal side: palatal CEJ to midline NPCEJ Total: bilateral palatal CEJ distance PCEJ	Coronal Coronal Coronal	Distance between the palatal CEJ of the maxillary first permanent molar in the cleft-side and the midline Distance between the palatal CEJ of the maxillary first permanent molar in the normal-side and the midline Distance between the bilateral palatal CEJ of the maxillary first permanent molars
5	Cleft side: vestibular CEJ to midline CVCEJ Normal side: vestibular CEJ to midline NVCEJ Total: vestibular CEJ distance VCEJ	Coronal Coronal Coronal	Distance between the vestibular CEJ of the maxillary first permanent molar in the cleft-side and the midline Distance between the vestibular CEJ of the maxillary first permanent molar in the normal-side and the midline Distance between the bilateral vestibular CEJ of the maxillary first permanent molars
6	Cleft side: jugular to midline CJUG Normal side: jugular to midline NJUG Total: jugular JUG	Coronal Coronal Coronal	Distance between the cleft-side intersection of the horizontal level of the lower cortical of the hard palate-maxillary tuberosity and the midline Distance between the normal-side intersection of the horizontal level of the lower cortical of the hard palate-maxillary tuberosity and the midline Distance between the bilateral intersection of the horizontal level of the lower cortical of the hard palate and the maxillary tuberosity
7	Cleft side: arch length CAL Normal side: arch length NAL Total: arch length AL	Panorama Panorama Panorama	Distance between the maxillary first permanent molar distal CEJ in the cleft-side and the midline Distance between the maxillary first permanent molar distal CEJ in the normal-side and the midline Distance between the maxillary first permanent molars distal CEJ

The panoramic view was meticulously reconstructed, by manually tracing the CEJ of the presented permanent teeth in an axial slice. Additionally, the line passing through the midpalatal suture in the transverse plane and panoramic reconstruction was carefully monitored in axial and sagittal slices.

### Measurement determination

The selected CBCT-based measurements were designed and developed by the authors based on maxillary anatomy, the FOV of our acquired CBCT scans, and previous research [23–25]. We based these measurements on the maxillary first permanent molars, as they were fully erupted by the time of the preoperative CBCT scan. We also explored various linear measurements in the premaxillary region; however, this was not feasible to apply due to the considerable deviation of tooth positions in the premaxillary area and the abnormal anatomy resulting from the cleft. The selected dentoskeletal measurements represent crucial regions within the maxilla, encompassing both dentoalveolar and skeletal aspects. Importantly, the chosen anatomical landmarks are not in direct contact with the cleft or the site of SABG.

Manual measurements were performed, using Romexis^®^ software version (4.6.0.R, Planmeca Oy, Helsinki, Finland). Maxillary dimensional changes were followed-up by comparing the same dentoalveolar and skeletal distances between the preoperative and postoperative CBCT scans for each patient. These specific distances were measured in sagittal, transversal, and axial slices, as well as panoramic reconstruction. Measurements were carried out by a trained dentist and an Oral and Maxillofacial Radiologist, both working at the department of Oral Radiology. Analysis was preceded by a calibration session on all study variables between the two observers. The measurements were performed separately in a dimmed room, utilizing a Medical Display (Eizo FlexScan MX190) screen. All landmark measurements for 20% of randomly chosen subjects were repeated twice by both observers, with a three-month interval to assess inter- and intra-observer reliability and ensure consistency.

### Statistical analysis

Statistical analyses were conducted using SPSS software (version 28.0.0; SPSS Inc., Chicago, IL, USA). The normal distribution of the data was confirmed by the Shapiro-Wilk normality test. To assess intra- and inter-observer reliability, the Intraclass Correlation Coefficient (ICC) test with a two-way mixed-effects model was applied [26]. Based on 95% confidence interval (CI) of the ICC estimate, reliability values: less than 0.5 suggest poor reliability, between 0.5 and 0.75 indicate moderate reliability, between 0.75 and 0.9 signify good reliability, and values greater than 0.90 reflect excellent reliability [27]. A paired t-test was employed to evaluate significant differences between the preoperative and postoperative measurements, irrespective of age and gender, with statistical significance set at *p* < 0.05.

For assessing the impact of age and gender, as well as their interrelation, a two-way analysis of variance (ANOVA) test was employed, with statistical significance set at *p* < 0.05. Patients were stratified into two distinct age cohorts based on their age at the time of the preoperative CBCT scan: a younger group consisting of 15 patients aged 7–8, and an older group comprising 32 patients aged 9–11.

## Results

A total of 47 patients, comprising 32 males and 15 females, were selected based on the specified criteria. Six patients were excluded due to poor quality CBCT scans, and two overaged patients were excluded. On average, CBCT examinations were performed 5.8 months before SABG and 6.6 months after SABG. Inter-and intra-observer reliability tests demonstrated excellent agreement ( = > 0.90), across all the study variables in both the preoperative and postoperative measurements Table 2.

**Table 2:** Intra-class correlation coefficient (ICC) with 95% confidence interval (CI) for all the study variables between the two observers

Preoperative Measurements	ICC	95% CI	Postoperative measurements	ICC	95% CI
ANS-PNS	0.93	(0.54–0.94)	ANS-PNS	0.98	(0.91–0.99)
CF	0.99	(0.96-1.00)	CF	0.97	(0.94-1.00)
PVD	0.95	(0.87-1.00)	PVD	0.98	(0.87–0.99)
PCEJ	0.98	(0.88–0.99)	PCEJ	0.96	(0.65–0.99)
VCEJ	0.98	(0.87–0.99)	VCEJ	0.99	(0.97-1.00)
JUG	0.98	(0.89–0.99)	JUG	0.98	(0.89–0.99)
AL	0.98	(0.41–0.99)	AL	0.93	(0.16–0.93)

**Fig. 4:**
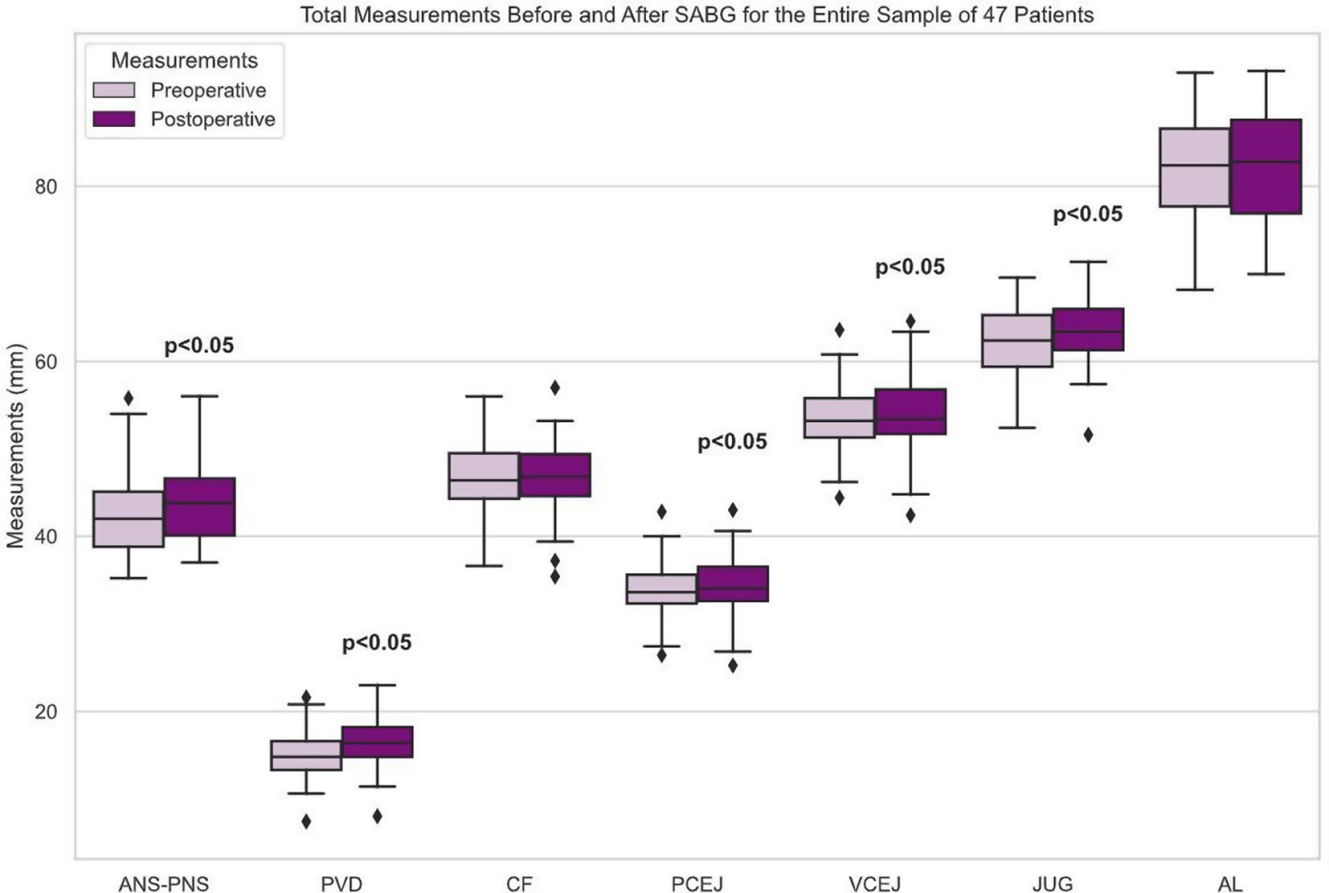
Box plots represent measurements taken before and after SABG, for the entire sample of 47 patients. The center lines indicate the medians, while the box limits represent the 25th and 75th percentiles. Outliers are depicted as dots. ANS-PNS, PVD, PCEJ, VCEJ, JUG (*p* < 0.05)

The mean age of patients at the time of the preoperative scan was 8.8 years, while it was 10 years at the time of the postoperative scan. Table 3 presents the descriptive statistics of mean linear maxillary dimensions for all patients in the preoperative and postoperative measurements. All linear measurements showed an increase, except for CF and AL, which remained almost the same. However, the increase was statistically significant in all skeletal-based measurements (ANS-PNS, PVD, and JUG) (*p* < 0.05) and two dentoalveolar-based measurements (PCEJ and VCEJ) (*p* < 0.05) when comparing measurements between the preoperative and postoperative scans for the entire sample, as illustrated in Fig. 4.

The two-way ANOVA test revealed that age groups did not have a significant influence on the study variables. However, gender had a substantial impact on the following transverse dimensions: CF, VCEJ, and JUG, with males displaying significantly larger dimensions (*p* < 0.05). Moreover, the interrelationship between age and gender showed an exclusive statistical significance in postoperative AL (*p* > 0.05), where younger males exhibited a greater arch length, as presented in Table 4. However, these differences should only be considered as indicative, as the groups are too small to draw any definitive conclusions.

**Table 3:** Mean ± standard deviation (SD) of linear measurements (mm) performed on the preoperative and postoperative CBCT data for the total sample (47 patients). The age represents the patient’s age at the time of the CBCT scan. A paired t-test was applied to detect any significant differences between the preoperative and postoperative measurements, represented by P-values

	Preoperative	Postoperative	*P*-values
Mean age	8.8 ± 1	10 ± 1	
Measures in mm	Mean± (SD)	Mean± (SD)	
ANS-PNS	42.4 ± 4.5	43.9 ± 4.6	0.00^*^
CCF NCF CF	23.5 ± 2.5 23.1 ± 1.9 46.6 ± 3.9	23.5 ± 2.6 23.1 ± 1.9 46.7 ± 4	0.3
PVD	15 ± 2.8	16.6 ± 2.9	0.00^*^
CPCEJ NPCEJ PCEJ	17.2 ± 2.1 16.7 ± 1.7 33.9 ± 3.3	17.4 ± 2.3 16.8 ± 1.7 34.3 ± 3.4	0.003^*^
CVCEJ NVCEJ VCEJ	27 ± 2.3 26.5 ± 2.1 53.6 ± 3.9	27.2 ± 2.5 26.7 ± 2.1 54 ± 4.2	0.007^*^
CJUG NJUG JUG	31.7 ± 2.2 30.6 ± 2 62.3 ± 3.9	32.5 ± 2.4 31.2 ± 1.9 63.7 ± 4	0.00^*^
CAL NAL AL	41.9 ± 4.1 40.3 ± 3.1 82.2 ± 6.4	41.9 ± 3.6 40.4 ± 3.4 82.3 ± 6.4	0.7

^*^ Statistically significant (*P* < 0.05) when comparing the postoperative measurements to the preoperative ones

**Table 4:** This table displays the p-values derived from a two-way ANOVA test conducted on all study variables. It illustrates the influences of age, gender, and the interaction between age and gender

Variable	Time	*P*-value (Gender)	*P*-value (Age)	*P*-value (Interrelation Gender and Age)
ANS-PNS	Preoperative	0.4	0.2	0.1
CF	Preoperative	0.04*	0.8	0.8
PVD	Preoperative	0.6	0.1	0.1
PCEJ	Preoperative	0.08	0.6	0.8
VCEJ	Preoperative	0.03*	0.4	0.5
JUG	Preoperative	0.03*	0.06	0.5
AL	Preoperative	0.2	0.4	0.06
ANS-PNS	Postoperative	0.4	0.08	0.07
CF	Postoperative	0.03*	0.6	0.9
PVD	Postoperative	0.7	0.7	0.1
PCEJ	Postoperative	0.06	0.6	0.9
VCEJ	Postoperative	0.03*	0.6	0.7
JUG	Postoperative	0.07	0.3	0.5
AL	Postoperative	0.2	0.7	0.03*

^*^ Statistically significant (*P* < 0.05)

When bilateral measures were compared, analysis revealed minimal and insignificant differences between the cleft side and the normal side in both transverse plane and panoramic reconstruction (*p* > 0.05).

## Discussion

The primary focus of the present study was not to assess the outcome of bone grafting nor the impact of SABG on growth. Instead, our aim was to explore the potential of adopting CBCT-based measurements as a method for quantitatively assessing maxillary changes and describing maxillary dimensions. While numerous studies have described dimensional changes in CL/P over the years, this study, to the best of our knowledge, is the first of its kind. It provides a 3D CBCT-based follow-up of maxillary changes post SABG over a designated timeframe. The selected linear measurements performed in pre- and postoperative CBCT examinations reflect maxillary bone changes in different planes: ANS-PNS (sagittal), PVD (vertical), and CF, PCEJ, VCEJ, JUG (transverse). The present study unveils that quantitative assessment of maxillary changes is feasible through our CBCT-based approach.

CBCT revealed statistically significant continuous expansion in all orthogonal planes of the maxilla when comparing the preoperative scans to the postoperative ones, irrespective of age and gender. Consequently, the null hypothesis can be rejected. These findings partially align with the outcomes of a recent study conducted on a healthy population when compared with patients within the same age range [25], where sagittal and vertical dimensions showed the largest changes during the follow-up. Nevertheless, the observed significant skeletal and dentoalveolar traverse expansion in our study was not noticed in the healthy population [25]. This finding was unexpected, considering prior research suggesting that transverse growth is mostly completed by the time of SABG [4]. A potential explanation for this discrepancy might be the influence of SABG in cleft patients. Further research is necessary to gain a deeper understanding of the impact of SABG on transverse growth, particularly since previous published studies have been limited to sagittal and vertical aspects of the maxilla through two-dimensional (2D) imaging.

Gender appears to play a role only in the transverse dimensions, with males having a wider maxilla within the studied FOV and age range. This finding partially aligns with the previous study conducted on healthy children, which suggested that male patients have significantly larger maxillary dimensions in all aspects than their female counterparts [25]. However, we observed comparable dimensions between males and females in the sagittal and vertical planes. It is important to interpret our findings with caution due to the short-term follow-up period and the small cohort size in this study.

Finally, when comparing cleft side to normal side measurements, insignificant differences were observed. This might be potentially explained by pre-graft orthodontic treatment performed in the transverse plane, as most of the preoperative orthodontic treatment aims to correct transverse collapse [28, 29]. Although pre-graft orthodontic treatment may have indirectly influenced the maxillary dimensions, it is important to note that no further orthodontic procedures were performed during our CBCT follow-up period. Therefore, any observed bone changes during this period might thus be explained by either surgery, growth, or a combination of both. However, it is crucial to acknowledge that more research is needed through prospective follow-up studies with larger samples to assess skeletal symmetry in unilateral CL/P, both prior and after SABG. Ideally, observations should be compared to normal bone growth in healthy children, if such a setup is ethically approved.

It should be noted that two exposure protocols were implemented in our study, resulting from variations in tube current and exposure time for the CBCT scans. Despite these different protocols, the image quality remained consistent in terms of structure visibility and landmark identification, as demonstrated by a recent study conducted on the same cohort [30]. Thus, the accuracy of our linear measurements was not affected in the lower resolution CBCTs.

As previously mentioned, numerous studies have been conducted to evaluate growth in unilateral CL/P patients and the effects of SABG; however, the findings remain controversial [31]. Some studies report inhibited maxillary growth following SABG [32, 33], while others have found no surgery-related inhibition [34–36]. It is crucial to note that all of these studies have primarily relied on 2D imaging through cephalometric landmarks. Cephalometric analysis can misinterpret dimensional changes by representing false landmarks movement, instead of the emerging distortion [37]. In fact, one major challenge in cephalometric analysis lies in accurately identifying landmarks in young CL/P cases, contributing significantly to cephalometric errors [13]. Apart from this, it is challenging to precisely measure 3D structures from single images or pairs of images in 2D. Hence, it is advisable to approach the outcomes of cephalometric studies on CL/P with caution [13]. In contrast, CBCT offers the advantage of providing a 3D view with no overlap of structures, resulting in distortion-free images with exceptionally high spatial resolution [38]. In CBCT imaging, the voxel dimensions are isotropic, signifying equal measurements in all directions (X, Y and Z) and consistent resolution throughout [38]. This characteristic of CBCT is advantageous, as it allows for precise measurements of specific structures in all three perpendicular planes. The existing body of research confirms the accuracy and reliability of linear measurements performed on CBCT images [39–41].

### Clinical relevance

The findings of this study demonstrated the clinical applicability of CBCT-based measurements for quantitively assessing maxillary changes after alveolar bone grafting in patients with unilateral CL/P. Future investigations could greatly benefit from utilizing our CBCT-based measurements to delineate the dimensions of the maxilla. This could be particularly insightful when applied to distinct ethnic cohorts or patients afflicted with bilateral cleft conditions. Such an approach could potentially unveil nuanced variations and contribute to a more personalized and effective treatment paradigm. This, in turn, can aid future studies in distinguishing between bone changes related to growth, bone grafting, or a combination of both. Understanding these maxillary changes is crucial in avoiding more complex interventions, such as orthognathic treatment [42, 43] and offers valuable insights that can optimize surgical, orthodontic and radiological intervention strategies. The utilization of CBCT is poised for continued growth in the foreseeable future, mirroring its upward trajectory thus far [38, 44]. We expect this trend to continue, fueled by the numerous advantages and technological advancements associated with CBCT technology, as well as ongoing research endeavors aimed at reducing radiation exposure during CBCT scans.

### Study limitations

Our study innovatively quantified the changes in maxillary dimensions. However, it is crucial to acknowledge that it lacked a control group, which hindered us from demonstrating how SABG affected growth in all dimensions, although that was not the primary aim of our study. Future studies could benefit from a larger cohort, enabling a more robust comparison between genders and ideally incorporating a control group.

The present sample comprised children treated in Stockholm. Although no data was collected regarding their ethnicity, this omission was due to ethical limitations. Moreover, our dimensional measurements were confined solely to the maxillary region, restricting the clinical implications to applications in maxillary skeletal diagnostics and treatment planning. Future studies could encompass broader ranges of both linear and angular dimensions across a more extensive craniofacial area including the mandible.

Finally, our study used CBCT scans exposed prior to and post-bone graft. A more extended follow-up period could have provided additional insights and is recommended for future longitudinal studies.

## Conclusion

The present study used a CBCT-based approach for quantitatively depicting maxillary changes in unilateral CL/P patients following SABG. Within the limitations of our study, this approach demonstrated continuous dimensional changes in all orthogonal planes. Pre- and post-operative maxillary dimensions were reported.

## Data Availability

The raw de-identified dataset used and/or analyzed during the present study is available from the corresponding author on reasonable request.

## Abbreviations

2D: Two-dimensional
3D: Three-dimensional
AL: Bilateral arch length
ANOVA: Analysis of variance
ANS-PNS: Anterior nasal spine-posterior nasal spine
CBCT: Cone Beam Computed Tomography
CEJ: Cementoenamel junction
CF: Bilateral maxillary first molar’s central fossa
CI: Confidence interval
CL/P: Cleft lip and/or palate
DICOM: Digital Imaging and Communications in Medicine
FOV: Field of view
ICC: Intraclass Correlation Coefficient
JUG: Bilateral jugular
PCEJ: Bilateral palatal cementoenamel junction
PVD: Palatal vault depth
SABG: Secondary alveolar bone grafting
VCEJ: Bilateral vestibular cementoenamel junction
